# Effects of Technique Asymmetry on 500 m Speed Skating Performance

**DOI:** 10.3390/bioengineering11090899

**Published:** 2024-09-07

**Authors:** Zimeng Liu, Meilin Ding, Masen Zhang, Bing Yu, Hui Liu

**Affiliations:** 1 School of Sport Science, Beijing Sport University, Beijing 100084, China; liuzimeng@bsu.edu.cn; 2 Tianshui Sports Bureau, Tianshui 741000, China; bsudml53@163.com; 3 Department of Physical Education, Northwestern Polytechnical University, Xi’an 710072, China; zhangmasenvip@163.com; 4 Center for Human Movement Science, Division of Physical Therapy, School of Medicine, The University of North Carolina at Chapel Hill, Chapel Hill, NC 27599, USA; 5 China Institute of Sports and Health, Beijing Sport University, Beijing 100084, China

**Keywords:** technique symmetry, speed skating performance, speed skating training

## Abstract

This study aimed to determine the effects of technique asymmetry on 500 m straight-track speed skating performance. We analyzed 20 elite skaters, measuring their joint angles, center of mass shift, and times and speeds during the gliding and push-off phases. The technique asymmetry index (ASI) was calculated for each parameter, and paired *t*-tests were used to compare bilateral asymmetry. Spearman correlation coefficients assessed the relationship between the ASI and both the average straight track speed and overall performance. Significant bilateral asymmetries in the knee, push-off, trunk, and hip angles were found in both male and female participants (*p* < 0.05). The male participants demonstrated a higher right push-off speed (*p* = 0.029) and a longer left gliding time (*p* = 0.048). Significant asymmetry was also observed in the lateral shift of the center of mass during each phase of the straight-track skating gait cycle (*p* < 0.001). No significant correlation was found between the ASIs and the overall performance (*p* ≥ 0.067). These findings indicate that while elite speed skaters demonstrated significant bilateral technique asymmetry in straight track skating, these asymmetries did not significantly impact their overall performance.

## 1. Introduction

Bilateral asymmetries of strength, flexibility, and movements in athletes affect their performance in sports. Running and swimming are sports in which techniques ought to be bilaterally symmetrical. Studies, however, showed that athletes in these sports have bilateral asymmetry in strength and flexibility, which commonly leads to technique asymmetry in these sports [1,2,3]. In running, technique asymmetry not only reduces performance [4] but also increases the risk of hamstring muscle strain [5] and tibial fatigue fracture [6]. In swimming, technique asymmetry affects the effective propulsion force generated by the left and right limbs, which has a significant negative effect on swimmers’ performance [7,8]. The adaptations to asymmetric movements lead to asymmetry in bilateral limb functions [9].

Speed skating is a sport in which skaters show apparent asymmetry in strength. A study demonstrated that the maximum joint torques that professional skaters could generate at the left hip and ankle joints were significantly greater than those generated at the right hip and ankle joints [10]. These hip and ankle strength asymmetries were most likely due to the skaters’ adaptations to their skating on the curve track. On the curve track, speed skaters had to push both their legs to the right so they could turn left quickly [11]. Compared to the right leg, the left knee flexion angle is smaller when pushing the ice, so greater strength is needed from the left hip and knee joint extensors [10]. Consequently, the left semitendinosus, rectus femoris, and lateral gastrocnemius have significantly greater strength compared to their right side counterparts [10].

Although bilateral strength and technique asymmetries are necessary for the performance of curve skating, they may have negative effects on the performance of straight track skating and, thus, the overall performance of skating. Studies showed that the average curve speed as well as the average straight track speed of speed skating are significantly correlated with performance [12,13]. Studies also showed that the gliding distance, the trunk flexion angle, the knee flexion angle, the push-off angle, and the shift of center of mass (COM) are important technique factors affecting the average straight track speed [12,14,15]. Understanding the effects of the asymmetry of these technique factors on the performance of speed skating is important for speed skaters to adjust their training programs to maximize their performance.

Technical asymmetry in speed skating not only affects performance on the straight track but is also associated with an increased risk of injury. Studies have shown that a skater’s left lower limb is overloaded [16], and significant asymmetry in bilateral gluteus maximus fatigue [17] may increase the risk of lower limb injury [16,17]. Studying asymmetry in speed skating and adjusting training accordingly are important not only for improving performance but also for preventing possible injuries in speed skaters.

The purposes of this study were to determine (1) the bilateral technique asymmetry of elite speed skaters in straight track skating and (2) the effects of bilateral technique asymmetry on skating performance. We hypothesized that in straight track skating, (1) speed skaters could have bilateral asymmetry in their trunk flexion angle, knee flexion angle, and push-off angle; (2) speed skaters could have bilateral asymmetry in the shift of COM; (3) speed skaters may exhibit bilateral asymmetry in speed and time during the gliding and push-off stages; and (4) technique asymmetry could be negatively correlated with the average straight track speed and overall performance of skaters.

## 2. Methods

### 2.1. Participants

A total of 10 male and 10 female elite speed skaters were selected and agreed to participate in this study. They were the top 10 finishers in the men’s and women’s 500 m speed skating competition of the 2020–2021 China National Speed Skating Championships (Harbin, 28 March 2021). For the male participants, the overall performance was 35.99 ± 0.33 s, with the best performance being 35.64 s and the worst performance being 36.42 s. For the female participants, the overall performance was 39.46 ± 0.60 s, with the best performance being 38.34 s and the worst performance being 40.45 s. The best performance of each skater in two 500 m speed skating competitions was used for analyses of performance on the straight track during the first lap.

### 2.2. Data Collection

Skaters’ straight-track skating movements were recorded using 4 time-synchronized video cameras (SONY FDR-AX700, SONY, Tokyo, Japan) with a resolution of 1920 × 1080 at a sampling frequency of 60 frames/s [18]. Considering camera positions relative to movement direction, a shutter speed of 1/500 s was used to obtain videographic images with a blur error of less than 10 mm. The four video cameras were set as two groups with two cameras in each group. One camera was set on the side of the track and the other on the front side of the track in each group. The calibration space of each group of cameras was 14 m long × 6 m wide × 2.5 m high with an overlap area of 4 m long. The total calibrated space was 24 m long × 6 m wide × 2.5 m high. The calibration space was 76 m in front of the starting line.

A suspended calibration system was used to define each calibration space before the competition. The calibration system included four calibration poles with six calibration points suspended on each pole [18]. Five markers were placed in the overlap area of two calibration spaces for defining the reference frame in data processing. The ice surface reference frame was defined with the *X*-axis pointing in the forward direction of skating, the *Y*-axis pointing to the left side of the track, and the *Z*-axis pointing upward. The skaters’ skating movements in the calibration space were recorded in the competitions.

### 2.3. Data Process

The video records of skating were digitized using an artificial intelligence markerless motion capture system (FastMove Inc., Dalian, China) to obtain two-dimensional (2D) coordinates of 21 critical body landmarks [18,19]. Digitized 2D coordinates from two cameras were synchronized using multiple critical events [20,21]. The critical events included blades landing on the ice and blades taking off from the ice. Three-dimensional (3D) coordinates of 21 body landmarks were obtained from synchronized 2D coordinates using the Direct Linear Transformation method [22]. The mean calibration error was less than 0.005 m. The raw 3D coordinates of body landmarks were filtered through the Butterworth low-pass filter with an estimated optimal frequency of 10 Hz [23].

The artificial intelligence markerless motion capture system was used to collect 3D coordinates of body landmarks in this study. The reproducibility of the 3D coordinates of body landmarks collected using this system was rigorously evaluated. The multiple correlation coefficients of repeatedly collected coordinate data were no less than 0.90 [18,19], which indicated that the data obtained using this system were highly reproducible.

### 2.4. Data Reduction

**Figure 1:**
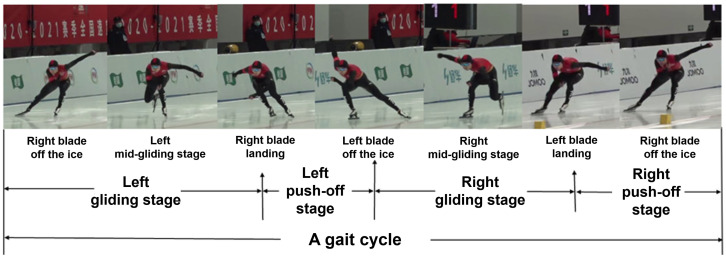
Movement-stage division diagram.

Displacements and the speeds of whole body COM, movement time, push-off angle, knee and hip flexion angles and range of flexion–extension motions (ROMs), trunk flexion angle, and flexion ROMs were calculated from smoothed 3D coordinates of body landmarks. A speed skating gait cycle on the straight track was defined as the duration between the two consecutive push-offs of the same side and consisted of a gliding phase and a push-off phase for each side (Figure 1). Taking the left side as an example, the left leg gliding phase started when the right blade was taken off the ice and ended when the right blade was placed on the ice. The left leg push-off phase began when the right blade was placed on the ice and ended when the left blade was taken off the ice (Figure 1). We calculated the gliding distance, time, and speed in the gliding phase and push-off phase on each side. We also calculated the COM movement distances in the forward–backward, left–right, and up–down directions during the gliding phase and push-off phase.

Trunk flexion angle was referred to as the angle between the longitudinal axis of the trunk and the horizontal plane. Hip flexion angle was calculated as the angle between the longitudinal axes of the trunk and the thigh. Knee flexion angle was defined as the angle between the longitudinal axes of the thigh and the calf. Push-off angle was defined as the angle between the longitudinal axis of the calf and the horizontal plane. The range of motion of joints was defined as the difference between the maximum and minimum values of the joint angles in a certain movement phase.

The bilateral asymmetry indexes (ASIs) [24] were calculated as follows:

(1) $$\mathrm{ASI}=\frac{\left|X_{R}-X_{L}\right|}{0.5\left(X_{R}+X_{L}\right)}\times 100\%$$

where X_R_ refers to the right leg parameter and X_L_ refers to the left leg parameter. We calculated the ASIs of joint angles at landing, push-off, and off-ice; ranges of joint motions; COM movement; gliding distance; speed; and time during the push-off phase and gliding phase. The greater the ASI, the greater the bilateral asymmetry of the given technique parameter.

### 2.5. Data Analyses

To test the first three hypotheses, paired *t*-tests were performed to compare bilateral technique parameters for male and female participants, and there were no multiple comparisons in this study. To test the fourth hypothesis, Spearman correlation coefficients were calculated between the ASIs of the participants’ technical parameters and both the average straight track speed and the finishing time of 500 m skating. Statistical testing results with a Type I error rate less than 0.05 were considered statistically significant. All data analyses were performed using SPSS Computer Program Package Version 26.0 (SPSS Science, Chicago, IL, USA). Effect sizes were calculated using Cohen’s d. An effect size less than 0.5 was considered small. An effect size greater than 0.5 but not greater than 0.8 was considered medium. An effect size greater than 0.8 was considered large.

## 3. Results

### 3.1. Basic Performance Data by Gender

The average straight track speed was 13.53 ± 0.21 m/s for male participants and 12.45 ± 0.25 m/s for female participants. The average straight track speed was significantly correlated to the overall performance for male and female participants (R^2^ = 0.442, *p* = 0.036 for male participants; R^2^ = 0.479, *p* = 0.026 for female participants).

### 3.2. Asymmetry in Joint Angles and COM Shift

For male participants, the right knee flexion angle at the right blade landing was significantly greater than the left knee flexion angle at the left blade landing (*p* = 0.004) (Table 1). The trunk flexion angle at the left blade landing was significantly greater than that at the right blade landing (*p* = 0.028) (Table 1). The left leg push-off angle was significantly greater compared to the right leg push-off angle (takeoff: *p* = 0.010; opposite-side landing: *p* = 0.020) (Table 1). No significant differences were observed in other bilateral joint angles and joint ROMs (Table 1 and Table 2).

For female participants, the left hip flexion angle at the left blade landing was significantly greater than the right hip flexion angle at the right blade landing (*p* = 0.040) (Table 1). The trunk flexion angle at the right blade landing was significantly greater than that at the left blade landing (*p* = 0.009) (Table 1). The trunk flexion angle at the left blade takeoff was significantly greater compared to that at the right blade takeoff (*p* = 0.048) (Table 1). The right knee flexion angle of the right leg at the right blade takeoff was significantly greater than that at the left blade takeoff (*p* = 0.032) (Table 1). The range of flexion–extension of the right knee during the right blade push-off phase was significantly greater compared to the left knee during the left blade push-off phase (*p* = 0.045) (Table 2). No significant differences were observed in other bilateral joint angles and joint ROMs (Table 1 and Table 2).

**Figure 2:**
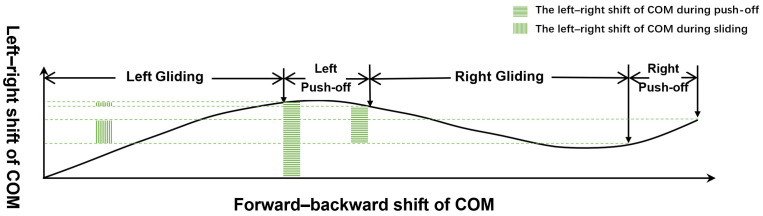
Asymmetry of left–right shift of COM.

The results of this study also showed that for both male and female speed participants, the left shift of COM during the left gliding phase was significantly greater than the right shift of COM during the right gliding phase (*p* = 0.001) (Table 3). Similarly, during the right push-off phase, the left shift of COM was significantly greater than the right shift during the left push-off phase (*p* = 0.001) (Table 3). Figure 2 is a schematic diagram illustrating the asymmetric left–right shift of the participants’ COM.

### 3.3. Asymmetry in Speed, Distance, and Time

For male participants, their right push-off speed during the right push-off stage was significantly greater than the left push-off speed during the left push-off stage (*p* = 0.029) (Table 4). The left gliding time was significantly greater than the right gliding time (*p* = 0.048) (Table 4). No significant differences were observed in bilateral gliding speed, push-off time, push-off distance, and gliding distance.

For female participants, no significant differences were observed in bilateral speed, distance and time of gliding, or the push-off stage (Table 4).

### 3.4. Correlation between ASI and Performance

**Figure 3:**
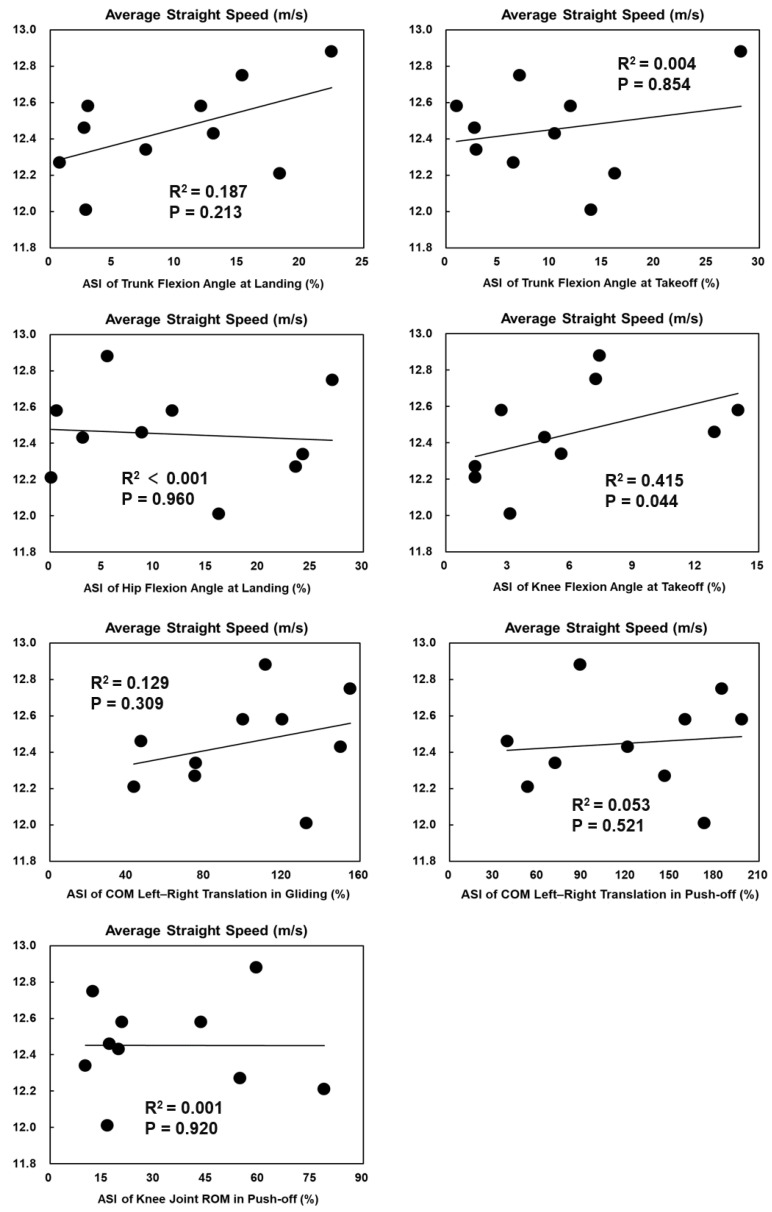
The Spearman correlation coefficient between the ASIs of technical parameters with significant bilateral asymmetry and the average straight speed in females.

**Figure 4:**
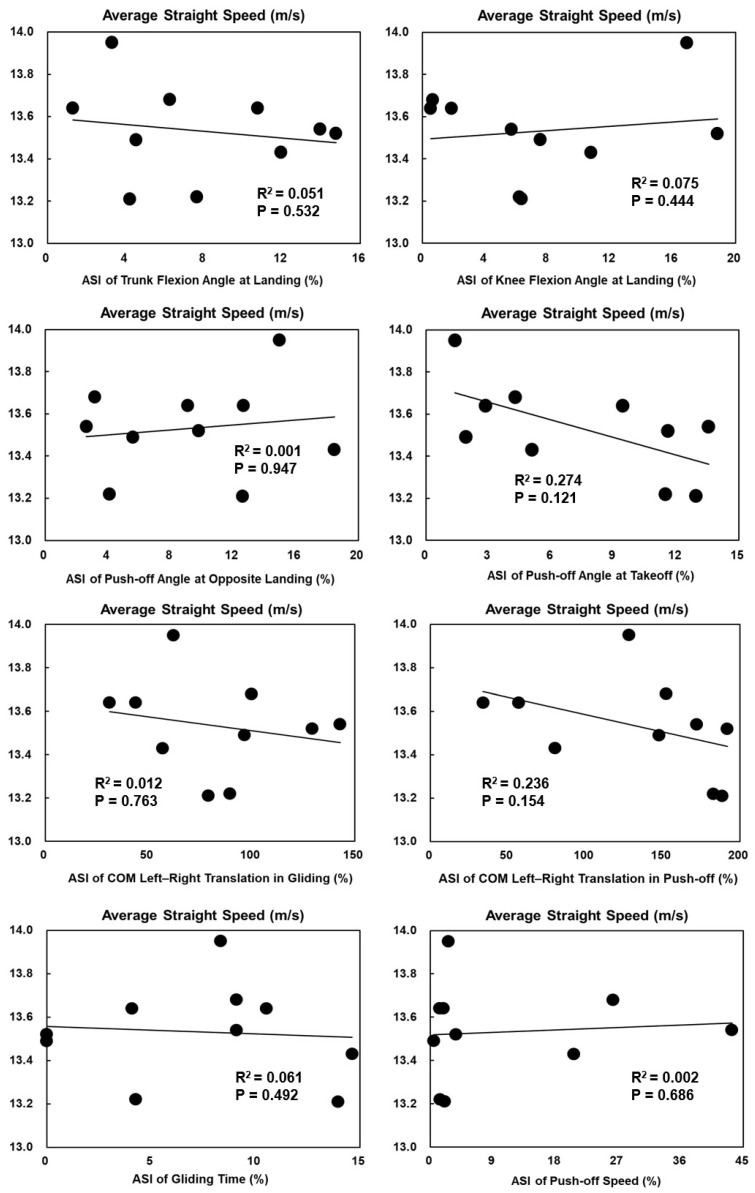
The Spearman correlation coefficient between the ASIs of technical parameters with significant bilateral asymmetry and the average straight speed in males.

**Figure 5:**
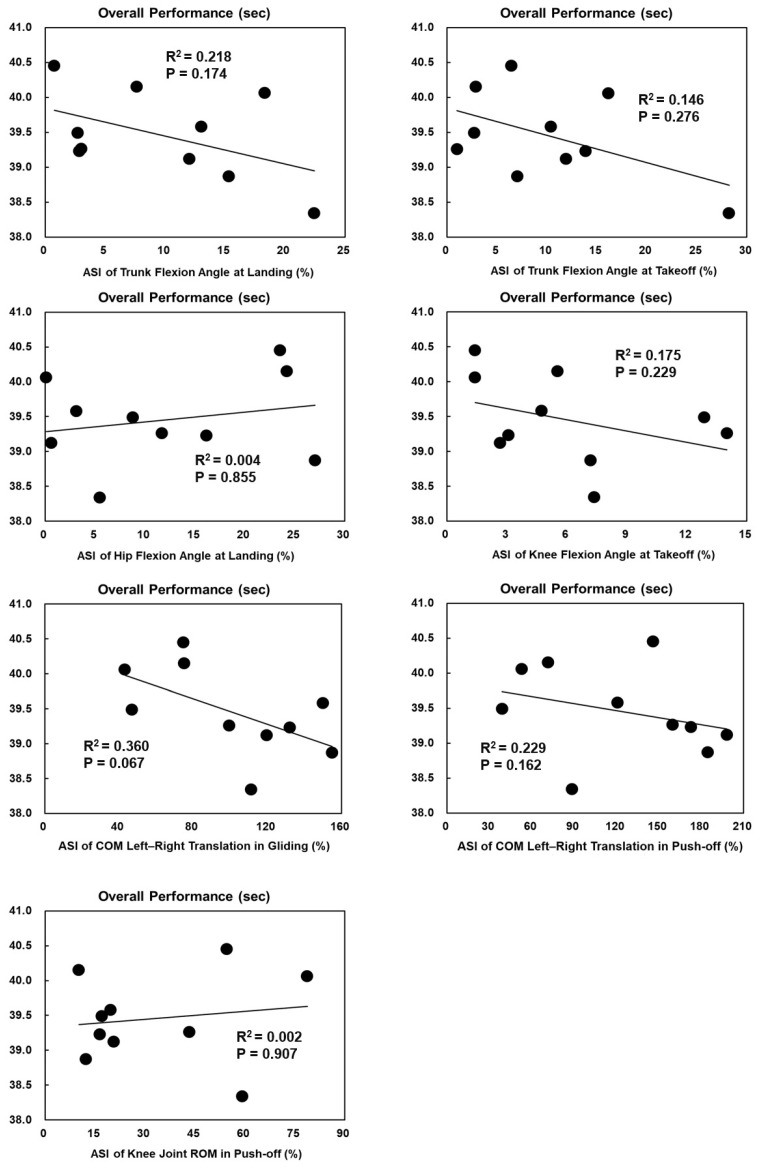
The Spearman correlation coefficient between the ASIs of technical parameters with significant bilateral asymmetry and overall performance in females.

**Figure 6:**
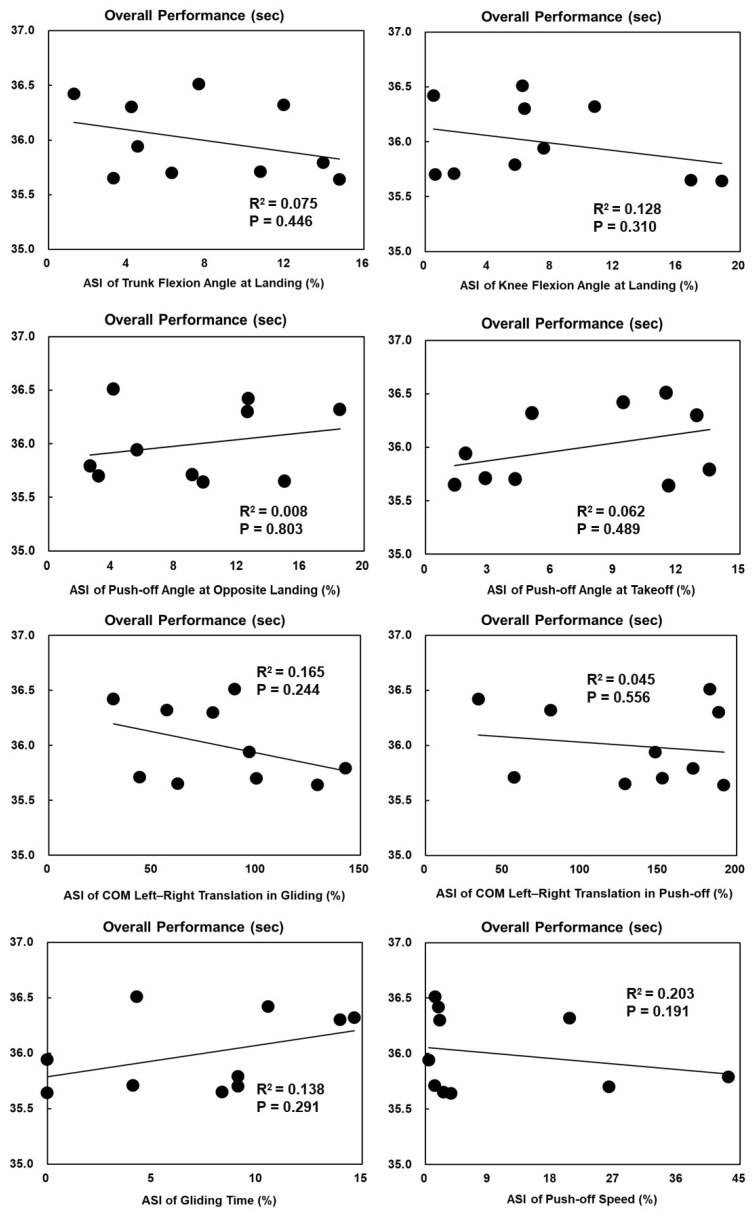
The Spearman correlation coefficient between the ASIs of technical parameters with significant bilateral asymmetry and overall performance in males.

The ASI of knee flexion angles at takeoff was significantly positively correlated with the average straight speed in female skaters (*p* = 0.044, R^2^ = 0.415) (Figure 3). Other ASIs of technical parameters with significant bilateral asymmetry had no significant correlations with average straight speed or overall performance (Figure 3, Figure 4, Figure 5 and Figure 6).

## 4. Discussion

The results of this study support our first hypothesis that speed skaters would have bilateral asymmetry in trunk flexion angle, knee flexion angle, and push-off angle in straight track skating. The male and female participants in this study showed significant asymmetry in different movements. The results of this study showed that male participants had a significantly smaller trunk flexion angle at the right blade landing compared to that at the left blade landing and significantly greater right knee flexion at the right blade landing compared to the left knee flexion at the left blade landing. The male participants’ left push-off angle was also significantly greater than the right push-off angle. These results indicate that, when landing on the right side, male participants’ knees exhibited a straighter position, and their trunk leaned more forward. When skaters pushed off with the right side, the angle between their blade and the ice surface was smaller.

These results demonstrated that male participants did indeed have left–right asymmetry in straight track skating. The results also showed that female participants had a significantly smaller trunk flexion angle at the left blade landing compared to that at the right blade landing and a significantly smaller ROM of right knee extension compared to that of the left knee during the push-off phases. Previous studies indicated that trunk flexion angle [25], knee flexion angle [25], and push-off angle [26] may affect speed skaters’ power output, which was related to their performance. Smaller trunk flexion angles were advantageous in reducing air friction, so skaters always tried to maintain their trunk in a horizontal position to minimize air friction [25]. Maintaining the trunk in a horizontal position limited the power generated by the hip joint, forcing the skater to rely on knee extension as the primary power generator [25]. Meanwhile, decreasing push-off angles increases lateral push-off force that may increase power output [26]. The technique asymmetries observed in the current study indicate that participants generated more power from their right side than from their left side, which is consistent with the findings of a previous study [27].

The results of this study support our second hypothesis that speed skaters would have bilateral asymmetry in shift of COM. The results of this study demonstrated that, for both male and female participants, the left shift of COM during the left gliding phase was significantly greater than the right shift of COM during the right gliding phase, and the shift of COM during the right push-off phase was significantly greater than that during the left push-off phase. These results demonstrate that the participants of this study had significant bilateral technique asymmetries in the shift of COM in straight track skating. Our study’s findings showed a greater shift of COM to the left compared to the shift of COM to the right, which might confirm the greater lower limb extension strength on the participants’ right side. Previous research linked skaters’ asymmetry in the shift of COM to bilateral push-off time and gliding time [15]. Our study’s findings showed that the shift of COM might also be related to skaters’ push-off speed. We found that the COM moved more to the left during the right push-off stage, which corresponded with faster right push-off speeds and subsequently longer left gliding times.

The results of this study support our third hypothesis that speed skaters exhibit bilateral asymmetry in speed and time during the gliding and push-off stages. The results of this study showed that male participants had significant bilateral asymmetries in the push-off speed and gliding time. A greater right push-off speed allows for a prolonged duration of gliding on the subsequent left side. For male participants, a greater right push-off speed suggests that the right lower limb could potentially possess greater push-off efficiency. This was consistent with a previous study that found greater extensor strength in the right leg during high-speed movements [28].

The participants of this study all demonstrated significant bilateral asymmetries in the selected technique parameters. The most likely explanation of these technique asymmetries is the long-term training adaptations on curve skating. Speed skaters rely on rapid and full extensions to generate power [29,30,31]. When speed skaters turn on curves, they tend to shift their COM toward the left leg to maintain balance and mainly use the right leg to provide power to maintain speed, which makes the right leg have more training load compared to the left leg. This long-term increased training load on the right leg results in greater strength in the right leg compared to the left leg, as shown in the literature [28].

The results of this study did not support our fourth hypothesis, which predicted a negative correlation between technique asymmetry and both the average straight track speed and overall performance. Instead, we found a significant positive correlation between the ASI of knee flexion angles at blade takeoff and the average straight track speed in female skaters. Specifically, a greater ASI of knee flexion angle at blade takeoff, with the right side angle being greater than the left, was associated with increased straight track speed. It was hypothesized that a larger right knee angle at takeoff indicated a more active right push-off, compensating for the imbalance caused by the stronger left leg. This asymmetry might have been an adaptation to the imbalance in bilateral force production, benefiting straight track speed but not necessarily affecting overall performance. Overall performance might have been influenced by a variety of other factors. Previous studies repeatedly showed that technique parameters were correlated with the performance of speed skating [12,14,15,25,26]. The results of the current study, combined with the literature, indicate that the effects of left and right leg movements on the performance of speed skating may be proportional. The effects of either leg’s movements, therefore, can be predictors of the performance of speed skating, but the difference in movements between the left and right leg are not. Although technique asymmetries do not appear to affect performance in speed skating, the strength asymmetries they cause may still be risk factors for lower extremity injuries [32]. Thus, further research may be needed to find ways to prevent injuries due to technique asymmetries.

The results of this study were limited to a relatively small sample size from the same country in the same speed skating event. The participants of this study were at a similar level. The training programs of these participants have many similarities. These limitations might prevent our study from showing the effects of technique asymmetries on the performance of 500 m speed skating. Further studies with a bigger sample size and in different speed skating events may be needed to confirm the results of the current study. The data of this study were collected in a real competition. The reproducibility of the results cannot be evaluated. Future studies are needed to confirm the results of the current study.

## 5. Conclusions

Speed skaters have bilateral technique asymmetries in straight track skating that are primarily evident in the knee flexion angle, trunk flexion angle, ROM of the knee joint, push-off angle, shift of COM, push-off speed, and gliding time. Only the asymmetry of the knee joint angle at takeoff has a significant positive effect on the performance of the straight track. Technique asymmetries have no effect on 500 m speed skating overall performance.

## Figures and Tables

**Table 1:** Bilateral asymmetry of joint angles at characteristic moments.

Gender	Technique Parameter	Moment	Right Side	Left Side	*p*-Value	Cohen’s d	ASI (%)
Male	Hip flexion angle (°)	Landing	60.5 ± 7.3	63.2 ± 9.9	0.202	0.31	12.17 ± 8.95
		Opposite-side landing	83.2 ± 7.9	84.4 ± 8.0	0.325	0.14	6.43 ± 6.44
		Takeoff	124.6 ± 10.6	126.4 ± 8.3	0.276	0.19	5.89 ± 4.46
	Knee flexion angle (°)	Landing	103.8 ± 8.8	96.2 ± 5.8	**0.004**	0.97	7.53 ± 6.34
		Opposite-side landing	122.9 ± 4.9	124.7 ± 4.7	0.184	0.35	3.59 ± 3.43
		Takeoff	163.0 ± 5.8	160.6 ± 8.6	0.185	0.31	4.02 ± 3.07
	Push-off angle (°)	Opposite-side landing	42.9 ± 3.2	45.9 ± 2.8	**0.020**	0.95	9.31 ± 5.39
		Takeoff	35.1 ± 2.6	37.3 ± 2.8	**0.010**	0.76	7.45 ± 4.82
	Trunk flexion angle (°)	Landing	26.1 ± 3.1	27.6 ± 3.6	**0.028**	0.43	7.87 ± 4.72
		Takeoff	31.9 ± 2.9	31.6 ± 4.6	0.399	0.08	8.86 ± 5.86
Female	Hip flexion angle (°)	Landing	58.4 ± 5.6	63.6 ± 6.5	**0.040**	0.86	12.07 ± 10.15
		Opposite-side landing	91.9 ± 10.8	97.8 ± 9.6	0.066	0.58	10.66 ± 8.28
		Takeoff	128.5 ± 7.1	130.1 ± 9.5	0.278	0.19	4.74 ± 4.27
	Knee flexion angle (°)	Landing	98.7 ± 7.4	100.2 ± 5.5	0.259	0.21	5.24 ± 4.79
		Opposite-side landing	131.8 ± 10.7	134.0 ± 13.5	0.320	0.17	10.08 ± 3.29
		Takeoff	162.4 ± 3.4	155.9 ± 10.5	**0.032**	0.80	6.03 ± 4.45
	Push-off angle (°)	Opposite-side landing	45.2 ± 3.1	46.0 ± 4.1	0.307	0.20	7.17 ± 6.48
		Takeoff	36.4 ± 2.5	36.4 ± 4.2	0.482	0.02	10.84 ± 6.14
	Trunk flexion angle (°)	Landing	28.9 ± 3.1	26.8 ± 4.0	**0.009**	0.57	9.81 ± 7.52
		Takeoff	29.7 ± 2.9	31.7 ± 4.5	**0.048**	0.52	10.05 ± 8.13

The bold in *p*-Value: Significant difference (*p* < 0.05) between left and right sides.

**Table 2:** Bilateral asymmetry of joint range of motion.

Gender	Technique Parameter	Stage	Right Side	Left Side	*p*-Value	Cohen’s d	ASI (%)
Male	Hip flexion–extension motion (°)	Gliding	27.3 ± 5.0	27.5 ± 10.4	0.472	0.03	31.45 ± 22.14
		Push-off	41.3 ± 9.5	42.0 ± 9.3	0.417	0.07	19.59 ± 11.37
	Knee flexion–extension motion (°)	Gliding	31.7 ± 6.0	32.5 ± 8.8	0.398	0.10	23.30 ± 16.24
		Push-off	40.4 ± 8.2	36.0 ± 8.6	0.089	0.50	22.75 ± 18.16
	Push-off motion (°)	Push-off	7.8 ± 4.9	8.6 ± 3.8	0.272	0.17	71.36 ± 105.33
	Trunk flexion–extension motion (°)	Gliding	8.7 ± 1.2	8.8 ± 2.7	0.458	0.04	22.77 ± 16.19
		Push-off	4.6 ± 2.0	5.9 ± 3.4	0.165	0.46	53.80 ± 32.89
Female	Hip flexion–extension motion (°)	Gliding	29.8 ± 11.7	37.3 ± 12.2	0.118	0.62	42.93 ± 40.33
		Push-off	36.7 ± 9.2	32.7 ± 12.7	0.116	0.36	24.05 ± 31.13
	Knee flexion–extension motion (°)	Gliding	34.8 ± 16.2	37.8 ± 12.3	0.325	0.20	52.62 ± 30.09
		Push-off	30.8 ± 10.5	25.4 ± 11.0	**0.045**	0.47	33.21 ± 23.95
	Push-off motion (°)	Push-off	8.8 ± 3.6	9.6 ± 3.2	0.284	0.23	40.45 ± 28.35
	Trunk flexion–extension motion (°)	Gliding	7.7 ± 2.7	6.7 ± 1.8	0.102	0.39	29.60 ± 22.80
		Push-off	3.1 ± 1.5	3.7 ± 2.4	0.235	0.26	44.43 ± 27.46

The bold in *p*-Value: Significant difference (*p* < 0.05) between left and right sides.

**Table 3:** Bilateral asymmetry of COM movement.

Gender	Stage	COM Movement (m)	Right Side	Left Side	*p*-Value	Cohen’s d	ASI (%)
Male	Gliding	Track length	4.923 ± 0.559	5.074 ± 0.325	0.140	0.33	7.85 ± 3.94
		Forward and backward	4.817 ± 0.529	5.009 ± 0.335	0.067	0.43	7.54 ± 3.45
		Left and right	0.181 ± 0.070	0.424 ± 0.081	**<0.001**	3.23	83.20 ± 35.91
		Up and down	0.018 ± 0.012	0.017 ± 0.013	0.456	0.06	90.46 ± 63.20
	Push-off	Track length	1.509 ± 0.254	1.494 ± 0.351	0.452	0.05	20.85 ± 15.29
		Forward and backward	1.500 ± 0.250	1.427 ± 0.387	0.298	0.23	24.35 ± 20.71
		Left and right	0.147 ± 0.068	0.026 ± 0.020	**<0.001**	2.42	133.46 ± 57.20
		Up and down	0.009 ± 0.006	0.009 ± 0.007	0.437	0.07	72.98 ± 67.53
Female	Gliding	Track length	4.435 ± 0.601	4.441 ± 0.392	0.488	0.01	12.21 ± 8.07
		Forward and backward	4.403 ± 0.578	4.380 ± 0.393	0.462	0.04	13.2 ± 38.90
		Left and right	0.128 ± 0.068	0.368 ± 0.083	**<0.001**	3.01	100.77 ± 39.96
		Up and down	0.022 ± 0.014	0.019 ± 0.015	0.344	0.17	80.53 ± 60.65
	Push-off	Track length	1.177 ± 0.297	1.135 ± 0.153	0.326	0.18	19.36 ± 18.10
		Forward and backward	1.168 ± 0.280	1.115 ± 0.137	0.293	0.23	20.37 ± 18.10
		Left and right	0.122 ± 0.042	0.025 ± 0.015	**<0.001**	2.93	123.34 ± 57.35
		Up and down	0.009 ± 0.007	0.010 ± 0.008	0.381	0.16	91.03 ± 64.52

The bold in *p*-Value: Significant difference (*p* < 0.05) between left and right sides.

**Table 4:** The bilateral asymmetry of speed, distance, and time.

Gender	Technique Parameter	Right Side	Left Side	*p*-Value	Cohen’s d	ASI (%)
Male	Gliding speed (m/s)	13.39 ± 0.42	13.29 ± 0.42	0.329	0.22	3.62 ± 3.00
	Push-off speed (m/s)	13.85 ± 0.30	12.64 ± 1.71	**0.029**	0.99	10.35 ± 14.73
	Gliding distance (m)	4.82 ± 0.50	5.01 ± 0.34	0.067	0.43	7.54 ± 3.45
	Push-off distance (m)	1.50 ± 0.24	1.43 ± 0.39	0.298	0.23	24.36 ± 20.71
	Gliding time (s)	0.36 ± 0.04	0.38 ± 0.21	**0.048**	0.53	7.40 ± 5.19
	Push-off time (s)	0.11 ± 0.02	0.11 ± 0.02	0.343	0.16	18.37 ± 13.93
Female	Gliding speed (m/s)	12.52 ± 0.39	12.28 ± 0.37	0.137	0.63	4.50 ± 3.01
	Push-off speed (m/s)	12.74 ± 0.60	12.46 ± 0.95	0.196	0.35	5.21 ± 7.10
	Gliding distance (m)	4.40 ± 0.61	4.38 ± 0.41	0.462	0.04	13.23 ± 8.90
	Push-off distance (m)	1.17 ± 0.30	1.12 ± 0.15	0.293	0.23	20.37 ± 18.10
	Gliding time (s)	0.35 ± 0.05	0.36 ± 0.03	0.377	0.12	10.81 ± 8.81
	Push-off time (s)	0.09 ± 0.02	0.09 ± 0.01	0.406	0.09	17.79 ± 18.37

The bold in *p*-Value: Significant difference (*p* < 0.05) between left and right sides.

## Data Availability

Pseudonymized datasets are available to external collaborators subject to agreement on the terms of data use and publication of results. To request the data, please contact Zimeng Liu (liuzimeng@bsu.edu.cn).
